# Collecting migrants' Facebook posts: Accounting for ethical measures in a text-as-data approach

**DOI:** 10.3389/fsoc.2022.932908

**Published:** 2023-01-09

**Authors:** Helena Dedecek Gertz

**Affiliations:** Department of Intercultural Education Research, Faculty of Education, Hamburg University, Hamburg, Germany

**Keywords:** internet research ethics, migrants' online groups, migrants' Facebook groups, text as data, topic modeling, contextual integrity

## Abstract

Based on the heuristics proposed by Helen Nissenbaum to assess ethical issues surrounding research using new technologies, this paper discusses the ethics of the collection and analysis of migrants' digital traces for academic research purposes. Concretely, this paper is grounded on an empirical research that applies a topic modeling approach to a large dataset of migrants' posts written on Facebook groups. After discussing the nine aspects proposed by Nissenbaum, the paper contends that while researchers strive to comply with ethical measures by, for instance, asking adequate questions and protecting the collected data, the lack of transparency of social networking sites is harmful to critical social sciences and can hamper findings that contribute to understanding migratory patterns and decisions.

## Introduction

The analysis of migrants' media use can produce valuable knowledge about decision-making and networks in migratory context. Methodologically, the interest in researching migrants' media use has added to the complexity of carefully handling migrants' data, and obtaining informed consent for research purposes. Accordingly, the ethics of collecting migrants' digital traces has been gaining attention, particularly among qualitative researchers (Leurs, 2017; Siapera and Creta, 2020; Sandberg et al., 2022b). Most of these ethical reflections agree that, because of the vulnerability of certain migrant populations, researchers need to go beyond procedural ethics and care for the safety and well-being of researched subjects.

Quantitative studies based on migrants' digital traces generate different problems relating to “profiling, informed consent, data sharing processes and ethical approval and data management procedures” (Mahoney et al., 2022b, p. 230). As there are fewer studies about migration applying topic modeling to social media data created by migrants, there are correspondingly fewer analyses on the ethics of collecting and analyzing such quantitative data. Mahoney et al. (2022b, p. 232) analyzed large textual datasets from migrants on Twitter, collecting only “explicitly public social media data”. They contend that ethical issues of such data collection become more intricate the more social media develops and the identification of public and private spaces becomes more complex (Idem, p. 235). Elsewhere, they carefully commend that datasets coming from migrant Facebook groups require consent, while collecting migrants' Twitter data would be closer to observing public behavior and therefore less problematic (Mahoney et al., 2022a; p. 339–340). A similar recommendation comes from Sandberg et al. (2022a).

As detailed on the section "Comparative evaluation based on studies using Facebook posts and a topic modeling approach,” studies using large amounts of Facebook texts tend to acknowledge that their methodological procedure can be liable to ethical critique, but do not analyze that ethical critique systematically. The most common solution for this dilemma tends to be to collect data that is interpreted as public or with fewer privacy constraints, such as posts from profiles with less privacy settings. By discussing the collection of large textual datasets posted by migrants on a social media platform, or Facebook more specifically, this paper systematically analyses the ethical decisions of an empirical research situation and argues in favor of research in digital humanities and social sciences. Against that background, this paper asks “how to justify the collection and analysis of migrants' digital traces for academic research purposes?” The discussion emerges from the procedures of collection and analysis of quantitative textual data from Facebook groups of migrants and aspiring migrants. Here, the outcomes of that empirical analysis are put in the background, giving way to a detailed reflection on the choices and consequences of the methodological decisions.

Following, first, the context and research design of the empirical base study is outlined. After that, the ethical issues and corrective measures are discussed guided by the heuristics of “contextual integrity” of information flow within new technologies proposed by Nissenbaum (2010) and as applied by Zimmer (2018). Although Nissenbaum presented her heuristics over a decade ago, they are based on broader concepts which make them comprehensive and abstract enough to be applied to different analytical situations. So much so that the heuristics are appropriate to discuss different empirical topics, such as data breaches from a dating app (Zimmer, 2018) and migrants' posts on Facebook groups. The paper summarizes the ethical boundaries of automated data collection, as encountered in the empirical base study conducted by Helena Dedecek Gertz and Florian Süßer (henceforth “we”, “our” or “the authors”) and presents our suggested measures to comply with migrants' data protection, adding up to the arguments for a reflexive and critical data collection based on ethics of care (Leurs, 2017). The central argument is that, although acknowledging that collecting textual data from social media users without their explicit consent is rightfully prone to critique, researchers, as a community, can care for migrants' anonymity throughout the process of research by making careful decisions to this end and by asking adequate research questions.

## Context of the data collection

The data that motivates the discussion here derives from a research project that aimed at identifying the roles of media in migratory pathways relating to education. Empirically, the project focuses on media uses of Brazilians who live in Germany or who aspire to do so. The project was based on a mixed-methods approach, consisting of a qualitative content analysis of interactions in migrant Facebook groups, qualitative interviews with participants of these groups, and a topic modeling of posts made in the groups. The ethical discussion in this paper derives from the empirical paper that applied the topic modeling approach to establish the prevalence of topics relating to education in debates among Brazilian migrants in Facebook groups. The outcome reveals that vocational education and training (VET) and language learning for certification purposes are the most relevant education-related topics debated among these migrants.

The background of that project is based on research that shows that, in the context of migration, formal education can represent a means to secure residence status, access the job market, and acquire certificates that contribute to building migrants' cultural capital (Waters, 2015). People who migrate to pursue educational pathways contribute to the transnationalization of educational institutions in the country of destination. Transnational education (TNE) is more often approached in research about higher education; nevertheless, migrants from families with low income and low educational attainment are also actors in TNE. Fürstenau (2019) and Carnicer (2019) have described how Brazilian women from such backgrounds migrate to Germany first as Au Pairs, then complete VET (which is usually remunerated in Germany), and thereby secure both employment and stable residence status.

Based on that background, the topic modeling (the empirical analysis that motivates this paper) had two assumptions. Based on the findings presented in the previous paragraph, one is that access to education can be a motivator for migration across socioeconomic classes, i.e., not only among migrants who can afford the pursuit of a university degree or educational exchanges abroad. The other assumption is that information exchanges through social media platforms are important for migrants' decision-making (Dekker et al., 2018; Richter et al., 2018). Although studies in this direction are mostly conducted among migrants who fled war and conflict using their smartphones' to evaluate the safest options to reach their countries of destination, information and communication technologies (ICTs) and exchanges with latent ties (Haythornthwaite, 2002) are relevant in other migratory contexts, such as those associated with educational aspirations (Jayadeva, 2020). Based on these two assumptions we contended that people cross borders, regardless of their socioeconomic background following educational projects, and that digitally mediated communication, particularly through social media, plays a role in decision-making for these projects.

Specifically for the Brazilian case, similar findings confirm the relevance of social media information exchange in contexts of migration. Brazilian migrants have been exchanging information on social media for at least a decade when the most used platform among them was Orkut (Schrooten, 2012; Oosterbaan, 2013). Nowadays, Brazilian migrant groups on Facebook groups have taken on that role in these online interactions (Foletto, 2018). Most of these studies on Brazilian migrants' on social media describe its uses for solving bureaucratic issues, job-seeking, and also for organizing social gatherings. Educational aspects remain under-commented, although education is a means to fulfilling migratory pathways and it can become part of migrants' life once they are established in the country and their children start attending school. While it is known that migrants who wish to pursue university degrees abroad use social media to facilitate that process (Jayadeva, 2020), the role of media use for achieving other educational levels, such as VET or schools for migrants' children, remains understudied.

Against that background, the quantitative textual analysis that motivated this ethical reflection reveals that topics relating to education, VET, and language-learning in particular, are among the most prevalent ones in information exchanges on Facebook groups of Brazilians in Germany. That conclusion was only possible through a topic modeling approach, which demanded the collection of quantitative textual data produced by migrants in the context of a social media platform. The following sections reflect on the ethics of collecting and analysing this data produced by individuals that are potentially vulnerable due to their legal status in Europe. Following, the methodological decisions that were ethically critical for this analysis are detailed; after that, we analyze our decisions based on the heuristics to guide ethical decision making in projects involving ICTs proposed by Nissenbaum (2010) and commented by Zimmer (2018). We conclude by arguing that, while researchers strive to comply with anonymization and data security, the lack of transparency from social media platforms can be harmful for critical, independent, and public-interest-oriented research, which in turn can impair the development of knowledge about social phenomena.

## Methodological decisions

In this section, we first discuss the choice of Facebook as a data source followed by an overview of ethical discourses in research about migrants' social media use and digital data collection. After that, we present our rationale for choosing Facebook groups adequate to answer our research question, explain our procedure of textual data collection, and argue in favor of a topic modeling approach to analyze the data.

### Creating a Facebook account for research

In migration research, Facebook has been mostly used as an empirical data source in qualitative approaches. Some accounts based on interviews about Facebook use among migrants (Leurs, 2014; Dekker et al., 2018) are exempt from a discussion such as the one we propose here, as informed consent can be acquired. As Leurs (2017) observes, however, researchers must still be careful with publishing digital traces of migrants, such as print screens or detailed information about certain media use patterns, even though interviewees themselves might have agreed to provide such data. That position is aligned with a way of arguing for a careful collection, management, and analysis also of quantitative textual data from Facebook, as “informed consent does little to protect participants” (Brown et al., 2016, p. 855) and researchers share the responsibility of caring for research participants' privacy and anonymity at all situations. Following such principles of care and transparency towards research participants, one of the authors created an account on Facebook.

The Facebook profile used for research was created using the researcher's real name, and with information identifying her as a researcher. Some friends and acquaintances added her and she joined five groups of her private interest (university and academic research related). She “liked” 76 public pages, most of them from organizations of Brazilians in Germany, but also some university profiles and a few of private interests. Finally, she joined 43 groups of Brazilians in Germany. Although she created this profile for research purposes, it is not a dummy account used simply to collect data, because she is clearly identified, with her name and picture, and with information signaling her as a researcher at Hamburg University. Her university e-mail and the website leading to the university's website, where she figures as a researcher, are also available on the page, as to make public other forms of contact with her (e-mail and telephone number on the faculty website) and to have some proof of her identity (the link to the website). Indeed, one person with whom the researcher got in touch through Facebook to ask for an interview for the qualitative part of the study, replied to her *via* email—and not through the Facebook chat where she sent out the message requesting the interview—to “be sure about the identity of the person who contacted me”, as the potential interviewee explained. Also, Facebook allows users to add free text to their profiles. In this space, she wrote her position at the university, the name of the project she works for, and informed that the profile was created for research purposes. This information was written in Portuguese, German, and English. Apart from this research-related information, there are traces of her personal interests (university groups and “likes” on pages) and information about her background (the town where she was born, where she currently lives, and her educational pathway).

### Contextualizing migrant Facebook groups selected for research

This section contextualizes the space of our data collection, namely Facebook groups gathering Brazilian migrants in Germany. First, we define these groups. Afterward, we describe the rationale behind the choice to analyze groups of Brazilians in Germany and the data collection procedure. Finally, we argue in favor of our decision to work with quantitative textual data in this context accounting for the research quality and ethics of our decisions. This section is already part of the contextual integrity analysis. Nissenbaum (2010) proposes nine points for the decision heuristics (see section “Discussing ethical decisions of research with migrants' textual data”). The second point of the heuristics is to identify the prevailing context of the information flow. As this section does exactly that, namely giving background information about the source of the data, this section substitutes the section “Prevailing context”.

Facebook groups of Brazilians in Germany gather registered users with similar interests, locations, jobs or professions, and aims. Some of these groups are public, meaning that their content can be seen by any other user logged on to Facebook. Other groups are private and might request users to fill up a form upon entry in order to be accepted by the administrators. Posts and comments on these groups are visible to all participants. These two types of groups can be found using Facebook's search tool and were included in this collection. There are “secret groups” for which one has to be invited to participate—there are none of these types in the dataset.

Although it has been argued (e.g., Naughton, 2022) that the use of Facebook has been declining, Brazilian migrant groups are still active and diverse, ranging from the general “Brazilians in Germany”, to the location-based groups, like “Brazilians in [German city]”, to specific groups like “Brazilian women in Germany”, to work-related groups, such as “Brazilian IT professionals in Germany”, “Brazilian Au Pairs in Germany”, aim-related groups “Ausbildung in Germany from Brazil”, and other interest groups “Gardening for Brazilians in Germany”. Finally, Brazilian migrants' fondness for social media groups is not new, as it has been reported already over a decade ago (Oosterbaan, 2010; Schrooten, 2012), nevertheless its uses for educational projects have not been studied. The paid VET programmes in Germany have the potential to attract migrants who are excluded from tertiary education in their countries of origin, as is the case of some Brazilians (Carnicer, 2019; Fürstenau, 2019)—hence the choice to focus our research on Germany as a country of destination. As to the decision to focus on Brazilians, the best-described case of migrants from low-income backgrounds pursuing tertiary education in a European country seems to be that of Brazilians. Similarly, the well-described case of Brazilians using social media to establish migrant networks in Europe (Oosterbaan, 2010; Schrooten, 2012; Foletto, 2018) lays the grounds for the choice of that particular nationality.

To select relevant groups for the empirical research, first Facebook was searched for the terms “Brazilians” and “Germany” (in Portuguese). After that, groups relating to migration were selected and these were once again filtered according to their level of interaction: using the information provided by Facebook itself, the author joined 43 “active groups”, i.e., groups with at least a thousand participants and three posts made in 1 week. From that total, 30 groups required participants to fill up a form upon entry to inform group managers about their interest in joining the group. We used the forms to inform the managers about our research interests, data collection, and anonymization measures. To perform the analysis, we used the structural topic modeling (STM) approach (Roberts et al., 2019), which allowed us to correlate the posts with the groups they came from. In this context, a topic is “a mixture over words where each word has a probability of belonging to a topic” (Roberts et al., 2019, p. 2). The outcome of the procedure reveals that among the seven most relevant topics, two are related to education, particularly to language learning and accessing vocational education and training in Germany. Across all groups, there is some mention of one of these topics, meaning that in a group gathering Brazilians in a specific city (but not explicitly related to education) or in a group gathering Brazilians who wish to pursue a degree in Germany, there is some mention of both education-related topics.

#### Automated textual data collection using a web-scraper

The empirical data for this analysis is textual and comes from posts and comments made by group participants. We solved the issue caused by the “APIcalypse” (Bruns, 2019) by automating our data collection using the WebDriver API *Selenium*, which allows us to automatically control a web browser. The scraper logs into the researcher's Facebook account and systematically goes through the groups that we could join. All posts, comments, and sub-comments were copied to a local file system. *Selenium* controls the web browser as if a human is sitting in front of it: all data obtained is exactly the same data available to the human Facebook user. No clicking behavior or friends list is collected, for instance. A human could do the same procedure, however with a much bigger investment of time and effort. Although we could have set up a Facebook Developer account that would allow us to use Facebook's API, the process using *Selenium* is not subject to Facebook's Graph API which includes rate limits. Summing up, automation simply sped up the process of data collection.

#### Reliability, reproducibility, and ethics in migrants' textual data collection

For the automated data collection, we considered whether and to what extent we would be violating terms of use from a giant social media company and if that would make us liable to a legal process. In that regard, we argue with those who stand for critical research (Hargittai and Sandvig, 2016; Bruns, 2019) and we support that independent data collection for social science and digital humanities research is rules-based and can comply with user privacy. Still, that does not solve the issue of the impossibility of gaining consent from all users when conducting such large data collection.

There are central differences between big techs' data extractivism and our procedures for data collection, storage, and analysis. These differences are due to the scientific interest, access, and use of the data. Regarding our data gathering, we as researchers using a Facebook user's interface with *Selenium* only had access to what other individual Facebook users also have. In that sense, we could control what information was collected. As for the storage procedure, the textual data was saved on a file shared only between the two authors of the empirical paper and that could only be accessed through a closed network. The applied “text as data” approach (Grimmer and Stewart, 2013) follows a standardized and, theoretically, reproducible methodology while complying with measures for data protection and having no financial profit. Nevertheless, because data from Facebook groups can be erased, entire groups or Facebook itself can cease to exist, and the platform can change its access rights, a reproducibility test is unlikely to result in the same *corpus*, hence such a test is not feasible.

It could be argued that there are other ways to research media use for educational projects on migration that would not demand a large textual data collection, therefore sparing this paper's discussion. That critique can also be directed to the nature of such quantitative textual data from social media: these Facebook posts are not connected to traits that characterize social positionality (e.g., socioeconomic background, educational attainment), which does not allow for an analysis that accounts for inequality and discrimination. In that sense, we agree with Leurs' critique (Leurs, 2017) that such procedures assume a “detachment from a discrete, knowable world” and tend to “naturalize the politics of knowledge production” (p. 134). However, because our empirical research question could only be answered with quantitative textual data focused on the prevalence of interactions involving education in migrant Facebook groups, the lack of such background information about group participants is not critical. Our topic modeling study is part of a larger project that includes a qualitative content analysis of the posts made in these Facebook groups and a digital ethnography, for which 30 group participants were interviewed. Following the logic that “all quantitative models of language are wrong—but some are useful” and that topic models need validation (Grimmer and Stewart, 2013, p. 269–270), apart from providing new research outputs by themselves, these qualitative approaches were used to validate findings from the topic modeling.

Finally, we do not treat data as “public” (Zimmer, 2010): we did not reproduce posts word-by-word, both as an ethical measure and because that was not useful for answering our empirical question. As a final compliance measure to counterbalance the impossibility of getting consent from group users and to reassure that anonymity is preserved, the empirical paper was presented to group managers and opened to their critique.

## Discussing ethical decisions of research with migrants' textual data

After contextualizing the study that generated this debate in the first section and describing methodological decisions in the previous section, we move on to discuss ethical decisions of our research design based on Nissenbaum's (2010) nine points for a contextual integrity analysis when using emerging media technologies for research. We also rely on ethical guidelines for internet research elaborated by the Association of Internet Researchers (AoIR) (franzke et al., 2020, p. 9-23).

Nissenbaum's nine points for contextual integrity are the following:

1. Describe the new practice in terms of its information flows (see our section Information flows).
2. Identify the prevailing context in which the practice takes place at a familiar level of generality, which should be suitably broad such that the impacts of any nested contexts might also be considered (section Prevailing context referring to section Contextualizing migrant Facebook groups selected for research).
3. Identify the information subjects, senders, and recipients (section Information subjects, senders, and recipients).
4. Identify the transmission principles: the conditions under which information ought (or ought not) to be shared between parties. These might be social or regulatory constraints, such as the expectation of reciprocity when friends share news, or the obligation for someone with a duty to report illegal activity (section Transmission principles and its subsections).
5. Detail the applicable entrenched informational norms within the context, and identify any points of departure the new practice introduces (section Detail the Entrenched Information Norms and its subsections).
6. Make a prima facie assessment: there may be a violation of contextual integrity if there are discrepancies in the above norms or practices, or if there are incomplete normative structures in the context to support the new practice (section Prima facie assessment).
7. Evaluation I: Consider the moral and political factors affected by the new practice. How might there be harms or threats to personal freedom or autonomy? Are there impacts on power structures, fairness, justice, or democracy? In some cases, the results might overwhelmingly favor accepting or rejecting the new practice, while in more controversial or difficult cases, further evaluation might be necessary (section Evaluation I).
8. Evaluation II: How does the new practice directly impinge on values, goals, and ends of the particular context? If there are harms or threats to freedom or autonomy, or fairness, justice, or democracy, what do these threats mean in relation to this context? (section Evaluation II).
9. Finally, on the basis of this evaluation, a determination can be made as to whether the new process violates contextual integrity in consideration of these wider factors (section Final determination). (Nissenbaum, 2010; p. 182–183).

In what follows, we address these items proposed by Nissenbaum thereby analyzing the ethical issues of collecting migrants' digital traces for academic research.

### Information flows

There are at least nine information flows on Facebook:

1. The first one is from the users to the company “Facebook” (clicking patterns, location, cookies).
2. The second one is the flow of information about the author who created the profile from the Facebook service to search engines and other non-users (if the profile is indexed on Google, for instance).
3. The third one is non-textual information from the profile owner to other users registered on Facebook (such as “likes” on profile pages and participation in groups).
4. The fourth one is from the private posts and friends list on the personal profile of the author who created the profile to their friends.
5. The fifth one is composed of posts written on private groups (groups, for which the administrator has to grant access to the requester), which can only be read by other group participants.
6. The sixth one is composed of posts written on public groups (groups, whose content can be seen by people who are not participating in it), which can be read by anyone who opens the group link.
7. The seventh one are replies to questionnaires elaborated by group administrators, as a requirement to be accepted in certain private Facebook groups.
8. The eight one are direct messages exchanged through the Facebook chat (which was used to contact potential interview partners for the qualitative study), i.e., a two-way flow between the profile owner and another person.
9. The ninth one are multiple-way direct messages exchanged among a closed group through the Facebook chat.

From this list, only the flows described on numbers 4 and 9 were not part of the interactions of the author who owns the profile, as she did not post anything on her private profile and did not send direct messages to multiple people. We did not create a dummy profile. Although the profile was created for research purposes, the owner was clearly identified on it and she did not try to conceal her intentions to group administrators when filling in questionnaires requesting to join the groups. By running the web-scraper, we did introduce a tenth information flow from the groups to our closed database, however our data did not include users' personal information. Finally, as our database is not public, the raw information flow is kept within the circuit of Facebook users (including the author who has a Facebook profile) and will be destroyed as soon as the research is concluded. One could argue that, once the analysis based on this data is published, there would be the eleventh flow of information toward the general public, nevertheless, that information is anonymized, filtered, and analyzed based on a specific research question. That flow is not of raw data; thus, it is a new circuit of information flow (from the publisher to its readers, etc.).

### Prevailing context

The prevailing context relates to the social context in which data is gathered (Nissenbaum, 2010, p. 149). For this paper, the prevailing context is that of Facebook groups of Brazilian migrants in Germany, as described particularly on section Contextualizing migrant Facebook Groups selected for research. This includes, among other aspects, the high level of use of Facebook among Brazilian migrants for networking and the opportunity of migrating to Germany to pursue tertiary education.

### Information subjects, senders, and recipients

In the context of our data collection, “information subjects” are the Facebook users who interact by writing posts with a question or a piece of information or by commenting on those posts in groups of Brazilians in Germany. People who participate in the groups but never had any interaction on them are not our information subjects. Within these information subjects, the senders are those who pose questions or share other kinds of information on the groups and those who reply to such questions. The recipients are all group members who read the interactions (either group participants or not, in the case of public groups). Because our dataset is not publicly available, we did not expand the role of recipients to the general public.

### Transmission principles

Transmission principles are rules that constrain the information flows (Nissenbaum, 2010, p. 145). In our case, there are three such sets of rules:

1. Because most of the information flows happen within a context controlled by a private company, Facebook's Terms of Service (ToS) are one of the regulators.
2. Because the data was collected in Germany, the General Data Protection Regulation (GDPR) from the European Union is a second regulator.
3. Because the collected data is part of an academic research, academic research ethics guidelines (e.g., franzke et al., 2020) apply.

Following, we comment on the central guidelines from these three sets of rules.

#### Facebook ToS

Facebook prohibits scrapping, but not manual data collection. We could have done the same procedure manually and acquired the same data however securing anonymity here would have been even worse because the person manually collecting the data would have seen what each group participant has written. The company's decision to prohibit scrapping after the Cambridge Analytica scandal is probably useful in constraining other companies to harvest and sell personal data that could be used for skewing public opinion on matters such as migration. However, that decision is harmful to social research (Sandvig, 2017; Bruns, 2019; Mancosu and Vegetti, 2020).

#### GDPR

GDPR recognizes that “by coupling information from registries, researchers can obtain new knowledge of great value … within social science, research on the basis of registries enables researchers to obtain essential knowledge about the long-term correlation of a number of social conditions such as unemployment and education with other life conditions”. As GDPR defines personal data as “any information relating to an identified or identifiable natural person” and as Facebook posts always appear associated with a profile, these texts could be interpreted as personal data. However, GDPR highlights that “information that identifies an individual … may be personal data if you are processing it to learn something about that individual or if your processing of this information will have an impact on that individual”. In this sense, the data we collected is in a gray zone: it is being used neither to learn something about an individual in particular nor to undertake actions that would have any foreseeable impact on an individual. In fact, we use these data to describe the social world, more specifically, digital information exchange relating to transnational education and migration.

Kotsios et al. (2019, p. 6–10) provide further instructions to assess the consequences of GDPR's seven principles relating to the processing of personal data in social media research. We present these seven principles and associate them with our case based on the comments by Kotsios et al. (2019) and on our experience with the empirical data described in the previous paragraphs:

1. Lawfulness, fairness, and transparency: Processing must be lawful, fair, and transparent to the data subject.
  Because Facebook itself does not provide “transparent data access to critical, independent, public-interest research” (Bruns, 2019, p. 1561), we cannot fully comply with this point. Facebook managers are likely aware that, even after closing their API, private companies still use web scrapers as well as researchers. The issue is that now the procedure is made opaque both to researchers and, as a consequence, to research subjects as well. We have taken the measures in our power to secure fairness and transparency as to our research purposes and data management standards.
2. Purpose limitation: Data must be processed for the legitimate purposes specified explicitly to the data subject when collected.
  Our data collection was conducted for public interest purposes, not for profit (Kotsios et al., 2019; p. 9-10), as is the case of private companies that also use web scrapers. The data we collected is going to be used solely for this research purpose and with our previously determined research question.
3. Data minimization: Only as much data as absolutely necessary for the purposes specified must be collected and processed.
  We had a defined timeframe for data collection (from December 2020 to January 2021) and we collected strictly data that was needed to answer our previously determined research question.
4. Accuracy: Personal data must be kept accurate and up to date.
  Once the collection timeframe was closed, the texts of collected posts were not edited content-wise. For the topic modeling analysis, we deleted stop-words (e.g., pronouns and conjunctions), diacritics (e.g., the letter “ç” or “ã”), and converted typical internet shortcut words into their traditional format (e.g., in Portuguese “as well” means “também” and is often written in online interactions as “tb” or “tbm”). This manipulation does not change the accuracy of posts' content. Instead, it serves to raise the accuracy of our topic modeling results.
5. Storage limitation: Personally identifying data can only be stored for as long as necessary for the specified purpose.
  The data will be destroyed as soon as the research project is finished.
6. Integrity and confidentiality: Processing must be done in such a way as to ensure appropriate security, integrity, and confidentiality (e.g., by using encryption).
  We have complied with this as described in section Methodological decisions.
7. ccountability: The data controller is responsible for being able to demonstrate GDPR compliance with all of these principles.
  As data controllers, we can comply with this measure.

As demonstrated, research based on social media texts can strive to comply with GDPR measures. However, the fact that social media companies like Facebook do not provide transparent information about their algorithm functionality and no longer facilitate data collection for academic research purposes puts researchers in a gray zone in regards to GDPR.

### AoIR guidelines

AoIR guidelines (franzke et al., 2020) are based on similar concerns as GDPR's, such as securing data privacy. However, AoIR guidelines are not laws, but rather stances for decision-making recommendations for scientific research. Instead of providing a panacea, AoIR guidelines emphasize researchers' ability to make sound judgments, which most importantly protect research subjects and researchers themselves (p. 23). These were the main guidelines we followed in our decision-making described at the beginning of the paper.

There is a clash: we are complying with AoIR, and we are in a gray zone of the ToS and GDPR. Our compliance rationale for these three constraints in information flow (ToS, GDPR, and AoIR) is aligned with the conclusions reached by Mancosu and Vegetti (2020), who claim that collecting textual data from Facebook pages can be “ethically and legally (GDPR) acceptable” (p. 9) but it might be in conflict with Facebook ToS.

### Detail the entrenched information norms

Such norms “describe the existing practices that prevail in a given context, encompassing the flows of information, transmission principles, and expectations of the actors involved” (Zimmer, 2018, p. 8). In our context, there are three groups of actors involved: migrants and aspiring migrants who participate in the groups, the group administrator(s), and the researchers. Because the interests of a company are divergent from those of these actors, Facebook is not accounted for here. Its entrenched information norms-related expectations can be interpreted according to the ToS described in the section Facebook ToS.

#### Migrants and aspiring migrants' expectations

As highlighted in section Methodological decisions, migrants have different reasons to join social media platforms and exchange information on these platforms. The entrenched information norms they have, however, are likely to be similar, namely that other humans will read what they have posted on the groups. There is an expectation that these other humans probably share similarities with them: be Brazilian migrants or aspiring migrants in Germany, have some relationship with Brazil and/or with Germany. There is also an expectation that the questions and other shared information on these groups will be replied to by these other humans who are likely to hold valuable information that can help solve the issue being asked about. As these groups are formed by over a thousand participants, it is not expected that all participants see the messages and reply to them, as well as there is an understanding that there are participants who are lurking in these groups (i.e., group participants who read the interactions but do not write). As these groups are highly populated and administrators cannot guarantee the identity of those who access groups, participants are likely to be careful with sharing personal information, and it is not uncommon that migrants anonymize themselves by not using their real names on social media. Finally, some public groups even accept the presence of company profiles that promote their services.

#### Group administrator(s)

Group administrators of public groups are likely to hold fewer entrenched information norms than administrators of private groups. The former is probably interested in having fewer moderation duties and possibly being recognized as the administrator of a large group highly relevant for the information exchange of a specific population (e.g., for the case of migrants in a specific town or migrants looking for education and employment in specific areas). For these administrators, which users participate in the group and for what purposes is probably irrelevant, as long as participants comply with their rules. The latter type of administrator is probably interested in having more control over who can access the group. Based on the questions from entry forms, their expectation is to filter participants who are likely to fuel disrupting discourses and those interested in using the groups to sell products and services. These administrators were informed about our interests as researchers.

#### Researchers

Our expectation was to observe migrants and aspiring migrants' textual interactions in a non-controlled situation. In doing so, we wanted to analyze the role of information exchanges in migratory projects related to education—or, how education projects relate to migration. More concretely, we expected to understand what migrants and aspiring migrants debate about education in these groups and to determine the relevance of education-related topics in these information exchanges. From the perspective of other group participants, as the researcher who owns the profile did not interact in the groups, she could be interpreted as a lurker.

As the author who created the profile is clearly identified on Facebook and as we did not harvest information such as location or other sensitive information that users may have made available on their personal Facebook profiles themselves, we did not disrupt users' expectation of being in a group with other people they do not know. In the eyes of these participants, we as researchers could be seen as any other lurker. As we did not expect to breach anonymity or to promote services or products, nor to do harm to participants, we complied with entrenched information norms of group administrators—and with ours as researchers. Furthermore, administrators of closed groups were explicitly informed about our expectations. Our results will also be shared with them in order to reassure them that we have secured that no group participant can be de-anonymized. Finally, as we are not making our data publicly available, we are not disrupting the informational norms of any actor.

### Prima facie assessment

Nissenbaum (2010, p. 182) contends that “a breach of informational norms yields a prima facie judgment that contextual integrity has been violated because presumption favors the entrenched practice”. Here, we land in a gray area. Considering that these groups are highly populated and therefore participants are careful with the information they publish in the group, that private groups' administrators were informed and allowed us to participate, and that we did not go against the expectations of public groups' administrators, we could argue that no informational norm was breached. Nevertheless, if we consider that the expectations of group participants were to exchange information, not to participate in a research project, then an informational norm was breached, particularly in relation to group participants and public groups' administrators. Still, our academic publication opens another information flow, because we have processed and analyzed the data, and therefore we are not sharing data that is part of the information flow described in part 4.1. In that sense, because there are two information circuits (the one among Facebook users only and the one derived from the publication of a paper based on the Facebook posts), and the data of one is not shared with the other, the situation is more complex and the entrenched information norms from the second circuit should also be assessed. Shortly, in that second circuit, peer-reviewers and the academic readership would probably like to have access to the data from the first circuit in order to assess the reliability of our analysis. However, if we do that, we would merge the two circuits of information and then doubtlessly breach contextual integrity by making our data public.

### Evaluation I

Considering that gray zone in relation to migrants' privacy and the breach of Facebook's ToS, we assume there is potential for a violation of contextual integrity and therefore, proceed to the first evaluation step to assess the gravity of the potential violation (Nissenbaum, 2010, p. 182).

Based on previous studies investigating migrants' use of social media (e.g., Dekker et al., 2018; Jayadeva, 2020), there is no evidence that such a topic of investigation might have caused harm to migrants. Researchers in this field have followed ethical procedures of anonymization and their research questions do not put the groups researched by them under any particular doubt or surveillance from authorities or other actors of migration. In that sense, there is no evidence that academic research about migration and digital media use has ever caused migrants to loose control over their information. Similarly, our proposal accounts for such security measures. In this sense, we are “doing no harm”, a primary ethical imperative (Fuchs and Unterberger, 2021).

### Evaluation II

The second evaluative step asks to assess how the new practice directly impinges on the values, goals, and ends of the particular context (Nissenbaum, 2010, p. 182). If we consider that, although one of us was participating in the groups not for the interest of exchanging information, but rather in analyzing it, then one could argue that we are not aligned with the goals of the context. However, as the person participating in the groups was completely identified, we treated the data carefully, we did not interfere in any discussions in the group, we did not collect private information, and users are not naive about participating in a group with over thousand unknown people, hence not sharing sensitive information and sometimes anonymizing themselves with aliases, one cannot say that we bluntly disrupted the values of the groups or of participants we have researched.

A model to assess factors affecting consent suggested by McKee and Porter (quoted in Elgesem, 2015, p. 15–16) and adapted to research with social media data by Elgesem (2015) helps to think about consent and anonymity within this gray area. What is helpful in that model is that it is based on scales, not on absolute statements. Assessing these scales can inform whether there is a requirement to obtain consent or if it is important to have consideration for consent (Elgesem, 2015; p. 18–19) and for the impossibility of obtaining it. The scales account for whether the data is rather private or rather public, and whether there are rather high or rather low issues involving topic sensitivity, degree of interaction of the researcher with subjects, and degree of vulnerability of subjects. For our case, the data collected are not personal communications between a small group of people, yet it was posted in a specific group of Brazilian migrants to which we had access, hence on this scale, we would still be in a gray area. Regarding topic sensitivity, we have a clear research question focused on transnational educational projects, which is not a topic of particular concern especially because we are not closely interacting with the researched subjects. Although remembrance of the experience of shattered educational aspirations and projects, for instance, can cause distress, our empirical research question focuses on what general topics are discussed in these groups. Furthermore, as we are not requesting group participants to access memories or share plans with us, i.e., we have a very low level of interaction with subjects, both our topic and our degree of interaction imply a comparatively low requirement of consent. Regarding the last factor, the subjects' vulnerability, we have to consider that we are researching migrants and aspiring migrants whose legal status is unknown to us. Again, we go back to our research question to judge whether there is a rather high or low requirement for consent. Differently from investigating migrants who use social media to inform their pathway to claim asylum and might have to resort to irregular practices for border crossing (Dekker et al., 2018; Fischer and Jørgensen, 2021), our research question relates to an issue that requires migrants to have a regularized status in the country, as without a residence permit, they cannot enroll in tertiary educational institutions. However, the situation is different for children and teenagers, who can access schools even though their parents might not have a regular migratory status in Germany. The possibility of inflicting direct psychological harm through our research topic is also low, as we did not interact with group participants. The possibility of inflicting indirect harm based on the outcomes of our research is also low, due to the focus of our research question in migratory projects involving education.

#### Comparative evaluation based on studies using Facebook posts and a topic modeling approach

This section comments upon other empirical research based on a topic modeling approach that also used data collected on Facebook. At the time of writing, to the best of our knowledge, there is no study using migrants' posts based on such an approach. The focus of this section is on other researchers' solutions and ethical justifications for data collection on Facebook regardless of the empirical topic of the studies. This overview reveals researchers' concern about the ethics of collecting such data but also an apparent avoidance to discuss these concerns in depth, perhaps either due to the earlier facilitated access to collecting Facebook posts (before the API's closure) or due to implicit perceptions of what public data is.

The discussion presented in this paper could have been spared if we had followed a less troublesome approach to data collection. An option could have been recruiting migrant Facebook users to participate in the research and requesting them to sign a consent form, as Verheijen and Stoop did for their linguistic analysis among Dutch speakers (Verheijen and Stoop, 2016, p. 249–258). They analyzed posts made only by these subjects who explicitly consented to have their posts collected. That would hardly be an option to research migrant Facebook groups. The reason for that is twofold: first, we could not force participants to post in the migrant Facebook groups, hence, if a participant did not post at all, we would have no data; and second, had we recruited participants with a high rate of posts, we would be cherry-picking the data since there is no evidence that most group participants have a high posting rate. Furthermore, even if we maximized or minimized demographic differences of such hypothetical participants, we would still have a non-representative sample because we do not know exactly what are the socio-economic characteristics of regular participants of these migrant Facebook groups. Hence, Verheijen and Stoop's (2016) solution would not suit our research aim.

Although also not related to migrants' use of Facebook, other methodological solutions closer to the one described in this paper reveal similar ethical concerns and contend that they cannot guarantee anonymization despite measures taken by the researchers (Merrill and Åkerlund, 2018, p. 340), while others do not focus on discussing the data collection and storage procedures (Puschmann et al., 2020; Amara et al., 2021; Heft et al., 2022). Most of these analyses are based on “public posts” or “publicly available profiles”, i.e., comments on Facebook pages of political parties and private organizations made by users who did not restrict who could view their posts. That decision seems to be implicitly presented as an ethical justification for collecting those posts. Furthermore, unlike ours, these other studies were conducted before Facebook closed its API, hence they do not mention the harms of that restriction to research.

The restriction posed by Facebook to social researchers is an obstacle in the analysis of social interactions, their causes, and consequences. Still, researchers keep using that platform and other platforms owned by the same company for their data collection, due to the social relevance it has reached. If earlier research, as described above, mentioned ethical concerns in a few sentences or left these concerns implicit, the closing of access to collect Facebook data has given impulse to reflect on ethics in practices of collecting digital traces (e.g., Bruns, 2019; Puschmann, 2019). That does not mean that the trade of “closing the access to relevant empirical data” for “elaborating on the ethics of collecting that data” was worth it: ethics of research using digital data had been already brought up before the closing of Facebook's API (e.g., Zimmer, 2010) and, as digitalization increases, there is no evidence that the ethical discussion in this field would have stopped. Nevertheless, this situation promotes advancements in the ethics' discussion at the same time that it sheds light on the power that a big-tech company has over academic research and researchers, as researchers might have to consider whether they make themselves liable for prosecution or decide to investigate topics through other methodological approaches even though using Facebook would be relevant.

## Final determination

The last point proposed by Nissenbaum (2010, p. 182–183) is the final determination as to whether there was a violation of contextual integrity and, if so, how grave is this violation toward whom, whether and how these violations are defensible. This final point is similar to our research question about how to justify the collection and analysis of migrants' digital traces for academic research.

We could have hired someone to copy and paste all posts and comments from Facebook, thereby complying with the ToS of not using an automated web scraper. The person doing this, however, would have had much more insight into who wrote what than an automated procedure. Facebook's decision of prohibiting web scraper might be well-thought to avoid companies profiling users and tackling the criticism toward the company after the Cambridge Analytica scandal, nevertheless, these policies are harmful to researchers who care for ethics and anonymity—as they can make themselves liable even though they have the best interest of not exposing vulnerable populations.

In this paper, we made transparent our data collection procedure and analyzed it in the light of ethical and legal frameworks. Along with Bruns (2019) and other critical researchers of digital media, we have added up the argument that such social media platforms occupy nowadays an important role in social phenomena and thus must “provide transparent data access to critical, independent, public-interest research” (p. 1561). For researchers studying migration and social media use, the lack of transparency of social media platforms implies a forced lack of control over the collected data. In turn, that impacts also migrants who could profit from critical views about digital media: research in this area can provide insights into reasons to migrate and decision-making processes supported by information exchanges on social media which can inform policies and support arguments in favor of migrants and diversity in media educational institutions, public discourses, and political spheres.

Based on the heuristic described in the previous eight points, the measures we took for the data collection through a topic modeling approach and its subsequent analysis do care for the anonymity of potentially vulnerable group participants. On the one hand, our decision not to make the data collected from the groups freely available further secures anonymity. On another hand, that puts us in a criticisable situation regarding the reliability of our data, as it cannot be shared. However, as securing data protection and anonymity of migrants who participate in these Facebook groups is more important in order to avoid harm, we decided to put more weight on that aspect than on the quality assessment of the academic community. In that sense, to some extent, securing the anonymity and data protection of vulnerable populations in academic research is a group commitment.

The collection of large textual datasets of migrants' digital traces for academic research purposes can be justified when researchers are invested in securing the collected data from anonymity breaches—by not collecting certain profiling data and by not creating another information flow by making their dataset available. The fact that academic research is guided by methodological and ethical guidelines and tends to be detached from financial profit also speaks in favor of the possibility of securing such datasets collected from migrant or vulnerable populations. In the unlikely case researchers had interest in selling their dataset for target advertising or political action against migration, for instance, the contextual integrity analysis described here would no longer be applicable and multiple contextual integrity violations would have been committed. Finally, although the procedures described here could be interpreted as in a legally gray zone, no involved parts were harmed in this data collection and analysis procedure. Therefore, such research is defensible when an appropriate research question is addressed and standards are followed, as researchers have already been doing (Mahoney et al., 2022a; Sandberg et al., 2022a).

## Data availability statement

The original contributions presented in the study are included in the article/supplementary material, further inquiries can be directed to the corresponding author/s.

## Author contributions

The author confirms being the sole contributor of this work and has approved it for publication.
